# COVID-19 and Politically Motivated Reasoning

**DOI:** 10.1177/0272989X221118078

**Published:** 2022-08-20

**Authors:** Allegra Maguire, Emil Persson, Daniel Västfjäll, Gustav Tinghög

**Affiliations:** Department of Management and Engineering, Division of Economics, Linköping University, Linköping, Sweden; Department of Management and Engineering, Division of Economics, Linköping University, Linköping, Sweden; Division of Psychology, Department of Behavioral Sciences and Learning, Linköping, Sweden; Department of Management and Engineering, Division of Economics, Linköping University, Linköping, Sweden; The National Center for Priority Setting in Health Care, Department of Medical and Health Sciences, Linköping University, Linköping, Sweden

**Keywords:** motivated reasoning, COVID-19, identity-protective cognition, science literacy

## Abstract

**Background:**

During the COVID-19 pandemic, the world witnessed a partisan segregation of beliefs toward the global health crisis and its management. Politically motivated reasoning, the tendency to interpret information in accordance with individual motives to protect valued beliefs rather than objectively considering the facts, could represent a key process involved in the polarization of attitudes. The objective of this study was to explore politically motivated reasoning when participants assess information regarding COVID-19.

**Design:**

We carried out a preregistered online experiment using a diverse sample (*N* = 1,500) from the United States. Both Republicans and Democrats assessed the same COVID-19–related information about the health effects of lockdowns, social distancing, vaccination, hydroxychloroquine, and wearing face masks.

**Results:**

At odds with our prestated hypothesis, we found no evidence in line with politically motivated reasoning when interpreting numerical information about COVID-19. Moreover, we found no evidence supporting the idea that numeric ability or cognitive sophistication bolster politically motivated reasoning in the case of COVID-19. Instead, our findings suggest that participants base their assessment on prior beliefs of the matter.

**Conclusions:**

Our findings suggest that politically polarized attitudes toward COVID-19 are more likely to be driven by lack of reasoning than politically motivated reasoning—a finding that opens potential avenues for combating political polarization about important health care topics.

**Highlights:**

- Participants assessed numerical information regarding the effect of different COVID-19 policies.
- We found no evidence in line with politically motivated reasoning when interpreting numerical information about COVID-19.
- Participants tend to base their assessment of COVID-19–related facts on prior beliefs of the matter.
- Politically polarized attitudes toward COVID-19 are more a result of lack of thinking than partisanship.

## Graphical abstract

**Graphical Abstract:**
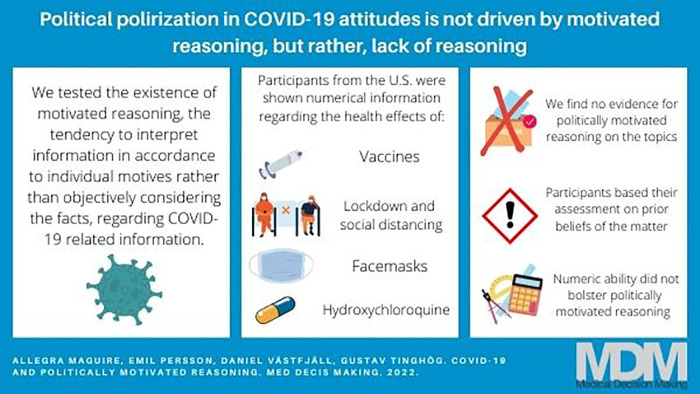
This is a visual representation of the abstract.

## Introduction

A striking development during the COVID-19 pandemic is how strong and influential the partisan divide has been on beliefs and public health behavior. Numerous polls and studies around the world have shown a clear partisan divide when it comes to beliefs about how serious the threat from the coronavirus is^1–5^ but also in terms of behaviors such as wearing face masks, practicing social distancing, and getting vaccinated.^1,6^ A geo-tracking study using data of 15 million smartphones per day across the United States showed that partisanship was the strongest predictor of practicing spatial distancing. The effect was also much stronger than for other indexes including COVID-19 spread and deaths in the geographical area.^7^ While the partisan divide is perhaps most prominent in the United States where Republicans and Democrats see COVID-19 very differently,^1,4,6,8,9^ the influence of political ideology on beliefs and public health behaviors has been demonstrated in countries around the world.^4,10–13^

What drives this pandemic partisan divide? The answer to this question can help countries and health organizations to develop science communication strategies that effectively increase compliance with public health initiatives and in the long run save lives.^14^ When it comes to health-protective behavior during the pandemic, views and behaviors have become strong signals of political and moral identity in the United States.^15^ Moral and political views are not easily changed or reshaped and can bias information processing.^16^ In fact, valued beliefs are sometime reinforced by new facts, even though this new information is not objectively supportive of held beliefs.^17,18^ Thus, the segregation in public health behavior may reflect a case of politically motivated reasoning, in which people evaluate information in a way to protect valued beliefs and political identity rather than objectively consider the facts and update beliefs.^19,20^

In the process of evaluating information or facts, politically motivated reasoning can be thought of as a tradeoff between desirability and accuracy, where people derive utility from maintaining valued beliefs, much like they derive utility from consumption and other types of behaviors in which they willingly engage.^21–23^ Although information avoidance and biased information seeking are strategies that also can help individuals to maintain valued beliefs,^22,24^ motivated reasoning occurs in the process of evaluating the information at hand.^25^ It should be noted that politically motivated reasoning is not the same as confirmation bias. The crucial difference is that politically motivated reasoning is about protecting ideological beliefs by selectively (dis)crediting facts to fit the identity-defining groups position on the matter, whereas confirmation bias merely is about selectively (dis)crediting facts to fit prior beliefs. Because politically motivated reasoning and confirmation bias often correlate, many incorrectly conflate them. Dan Kahan writes,

Someone who engages in politically motivated reasoning will predictably form beliefs consistent with the position that fits her predispositions. Because she will also selectively credit new information based on its congeniality to that same position, it will look like she is deriving the likelihood ratio from her priors. However, the correlation is spurious: a “third variable”—her motivation to form beliefs congenial to her identity—is the “cause” of both her priors and her likelihood ratio assessment.^19^

The cognitive route to motivated reasoning can broadly be conceptualized as either motivated reasoning as analysis or motivated reasoning as feeling.^26,27^ Motivated reasoning as analysis refers to the idea that identity-protective cognition is primarily driven by analytical, system 2 processing, in which people with a high cognitive ability are better equipped to reason their way around information that threatens valued beliefs. On the contrary, motivated reasoning as feelings refers to the idea that identity-protective cognition is primarily driven by intuitive, system 1 processing, in which people base their assessment of information on emotional cues to feel good. Thus, it is possible to protect valued beliefs both by thinking hard and by not thinking at all (i.e., motivated lack of reasoning).

The impact of politically motivated reasoning when processing information has been shown in many areas of public policy. In the US context, Democrats have been found to be more likely to trust scientific reports on climate change than Republicans,^28^ and Democrats were more likely to correctly interpret numerical information showing that banning guns decreased crime rates, whereas Republicans were more likely to correctly interpret information showing that banning guns increased crime rates.^29^ Similarly, in a Swedish context, globally oriented people were more likely to correctly interpret information showing that immigration decreased crime rates and vice-versa for nationally oriented people.^26^ Other policy areas in which politically motivated reasoning has also been established include performance of the national economy,^30^ beliefs about Iraq’s possession of weapons of mass destruction,^31^ the safety of vaccinations,^32,33^ the public health dangers associated with global warming,^34^ and the effects of different health care reforms.^35^ For a review of the literature, see Tappin et al.^36^

In the context of the COVID-19 pandemic, Pennycook and colleagues^4^ found that COVID-19 skepticism in the United States was strongly correlated with preferred news outlets, suggesting that the partisan divide may be driven by differences in information environments. In this study, we extend this research by exploring if the polarization is driven by differences in how people interpret the same information. Thus, the objective of this study was to explore politically motivated reasoning when participants assess information regarding COVID-19. We carried out an experimental online study on a sample from the United States. In line with previous research on partisan beliefs, we predicted that Republicans and Democrats would interpret numerical information about COVID-19 more in line with commonly held political views for the respective party, also when controlling for relevant individual differences, such as numeric ability and engagement in conspiracy beliefs.

## Methods

Sample size was determined in advance, and analyses were conducted only after data collection was complete. We report all conditions run and measures collected. The preregistration, materials, and data can all be accessed through the Open Science Framework (https://osf.io/wz3bv/). Informed consents were collected for all participants.

### Participants and Setting

A total of 2,227 English-speaking participants from the United States were recruited from CloudResearch^37^ to participate in an online experiment. Data were collected between October 27, 2020, and October 31, 2020. Thus, it was collected before the presidential election in the United States. In accordance with our preregistration, we excluded participants who failed the multiple attention checks (*n* = 681). We additionally excluded participants who finished the study in less than 2 min, since it was impossible to assess the materials in the experiment in such brief period (*n* = 46). Thus, the final sample consisted of 1,500 participants (38.7% males, 60.9% females; mean age = 40.9 y). Of these, 40.6% of the participants referred to themselves as Democrats, 34.0% as Republicans, 21.8% as Independents, and 3.6% as other or no preference. The experiment lasted on average 12.16 min, and participants received a flat fee of $1.70 as a compensation for their time. The experiment was programmed in Qualtrics.

### Materials and Procedure

**Figure 1:**
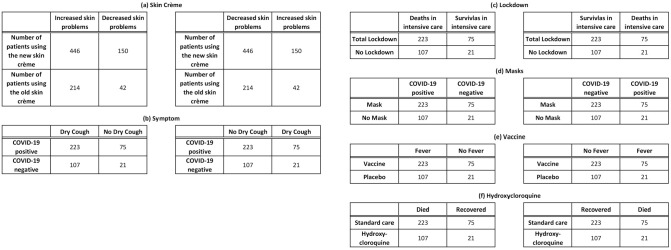
Experimental scenarios. Each scenario presented 2 versions with reversed effect obtained by switching columns in one contingency table. On the left, scenarios (a) and (b) are designed to be politically neutral and nonpolarizing. Scenario (a), skin crème, represents the main control condition since it is the only one not related to COVID-19. On the right, scenarios (c), (d), (e), and (f) are both related to COVID-19 and designed to be politically polarizing. Complete materials with the description of the scenarios and with behavior-feelings questions can be found in the supplementary materials.

Participants were randomly assigned to one of five conditions. Each condition contained two scenarios including tables providing numerical information of a treatment in fictitious studies about which the participants were asked to form judgments regarding their effectiveness. All participants faced a control scenario (skin crème) and one randomly selected COVID-19 scenario. The order of the scenarios was randomized. All scenarios were created in two versions, randomized between participants, with reversed columns in the contingency tables to control for the direction of the effect (see Figure 1).

Each scenario followed a similar structure. After reading a short text describing the scenario, the participants saw a 2-by-2 contingency table with the outcome of the study. They were asked to respond about the conclusions of the study. To correctly solve the problem, participants should compare the ratios between the numbers presented in each row. We implemented a total of six different scenarios: four politically polarized COVID-19 scenarios (masks, lockdown, vaccine, hydroxychloroquine), one COVID-19 scenario designed not to be politically polarizing (symptoms), and one control condition with a neutral scenario (labeled “skin crème”) designed to be unrelated to political orientation and to the pandemic.

#### Numerical task scenarios

Figure 1 shows all the scenarios employed. All scenarios had an alternative version that showed the inverse effect. The imputed numbers were the same as in Kahan et al.,^29^ but since participants responded to two scenarios, the values in the skin crème scenario were doubled. The control skin crème scenario was the same as in Lind et al.^26^ Participants read about a fictitious study testing the effects of a new skin lotion. In “skin increase,” the data from the fictitious study showed that using the new skin cream increased skin problems, and in “skin decrease,” the data instead showed that using the new skin cream decreased skin problems.

The COVID-19 scenarios were created around relevant topics that were the object of the ongoing “coronavirus infodemic” and that represent a threat to public health. In the “lockdown scenario,” participants were shown data from a fictitious study in which deaths in intensive care facilities were confronted in two districts of a country that differed in the employment of a total lockdown. In “lockdown-increase,” the data from the fictitious study showed that cases in intensive care facilities increased following lockdown, and in “lockdown-decrease,” the pattern was the opposite (i.e., cases decreased following lockdown). Since various protests against the stay-at-home orders were linked to right-wing groups,^38^ we expected this scenario to be politically oriented. We hypothesized that a larger proportion of Republicans than Democrats would provide a correct response to the “lockdown-increase” scenario and that the pattern would be the opposite for the “lockdown-decrease” scenario.

The “mask scenario” involved whether face masks can effectively hinder people from getting COVID-19, providing the numbers of people who tested positive or negative for people who used or did not use the dispositive. In “mask-increase,” the data from the fictitious study showed that using face masks increased the risk of getting COVID-19, and in the “mask-decrease,” scenario, the data instead showed that using face masks decreased the risk of getting COVID-19. Donald Trump’s disposition toward face masks is controversial, since the president declared to be favorable toward their use but appeared publicly without wearing one in multiple public occasions and spread skepticism over the effectiveness of face coverings.^39^ We hypothesized that a larger proportion of Republicans than Democrats would provide a correct response to the “mask-increase” scenario and that the pattern would be the opposite for the “mask-decrease” scenario.

In the “vaccine” scenario, participants were told that scientists were able to develop an effective vaccine for COVID-19 but were assessing possible negative side effects. Data were presented on side effects in contrast with a placebo. In “vaccine-increase,” the data from the fictitious study showed an increased rate of negative side effects due to the vaccine, and in “vaccine-decrease,” the data instead showed a decreased rate of side effects following vaccination. The public opinion toward the introduction of a vaccine for COVID-19 within the end of 2020 has been divided. For instance, during a CNN interview, the Democratic vice-presidential nominee Kamala Harris showed skepticism regarding taking a coronavirus vaccine herself if put out by the Trump administration.^40^ Attitudes toward a future vaccine seemed to be polarized based on political orientation.^41^ We hypothesized that a larger proportion of Democrats than Republicans would provide a correct response to the “vaccine-increase” scenario and that the pattern would be the opposite for the “vaccine-decrease” scenario.

The “hydroxychloroquine” scenario compared the effectiveness of the homonymous prophylaxis compared with standard care in hospitalized COVID-19 patients. In “hydroxychloroquine-increase,” the data from the fictitious study showed increased mortality rates for hospitalized COVID-19 patients treated with hydroxychloroquine, and for “hydroxychloroquine-decrease,” the data instead showed decreased mortality rates following hydroxychloroquine treatment. Hydroxychloroquine, which is a drug usually employed in fighting malaria, was endorsed by the leader of the Republican Party, Donald Trump, as a treatment for COVID-19.^42^ We hypothesized that a larger proportion of Democrats than Republicans would provide a correct response to the “hydroxychloroquine-increase” scenario and that the pattern would be the opposite for the “hydroxychloroquine-decrease” scenario.

Finally, the “symptom” scenario, which represents a control condition, regarded the possibility of differentiating COVID-19 patients from seasonal influenza patients and was intended to be nonpolitically oriented. In “symptom-increase,” the data from the fictitious study showed an increased rate of dry cough in COVID-19 patients compared with influenza (COVID-19–negative) patients, and in “symptom-decrease,” the data instead showed a decreased rate of dry cough in COVID-19 patients. We hypothesized that there would be no systematic difference in the proportion of correct responses between Republicans and Democrats in this scenario.

#### Political orientation

Political orientation, which represents a polarizing factor in the experimental design, was measured through two self-report scales: a continuous bipolar scale, from strong Democrat to strong Republican, which allowed us to quantify the strength of the political identification. We also included a “does not apply” option. The categorical measure asked participants what they usually consider themselves as: 1) Republican, 2) Democrat, 3) independent, 4) “other,” or 5) “no preference.” The two political polarization questions also operated as an attention and consistency check. We excluded participants who responded inconsistently to the two politically polarizing questions. For example, subjects who stated that they were Democrat on the categorical question but indicated that they leaned toward Republican in the other were excluded. In the analyses, we used responses to the categorical variable as our main politically polarizing variable. However, we conducted robustness checks using the continuous scale, and the results did not change in any substantial way.

#### Measures of individual differences

In addition to demographic variables, we collected data on individual differences in numeric ability and conspiracy beliefs. Previous studies have argued that the effect of politically motivated reasoning is exacerbated by cognitive sophistication and numeric ability, as people with high reasoning capacity will use that capacity selectively to process information in a manner that protects their own valued beliefs.^27^ A recent preregistered replication did, however, fail to find evidence in support of this pattern.^43^ Moreover, engagement in conspiracy theories has also been linked to motivated reasoning.^44^ Thus, we wanted to control for these measures. Numeric ability was measured following Lind et al.,^26^ using 6 items coming from Schwartz et al.,^45^ the Berlin Numeracy Test,^46^ and the Cognitive Reflection Task.^47^ This combination has previously been found to have good validity in measuring numeracy.^48^ To measure engagement in conspiracy beliefs regarding COVID-19, we used the scale from Biddlestone et al.,^44^ a 10-item, 7-point Likert-type scale. A complete list of the measures and all items is available in the Supplementary Materials.

### Statistical Analyses

In our main analyses, we first tested for politically motivated reasoning in each COVID-19 scenario by comparing the difference in the proportion of correct responses between Democrats and Republicans, as binary measure, across the 2 versions. For example, in the mask scenario, we calculated the difference in the proportion of correct responses between Democrats and Republicans for the “mask-increase” scenario, and we then tested if this difference was significantly larger (or smaller) than the corresponding difference between Democrats and Republicans in the “mask-decrease” scenario. We followed up with robustness analyses of these results using logistic models, controlling for age, gender, and education. We then analyzed all COVID-19 polarized scenarios (lockdown, mask, vaccine, hydroxychloroquine) jointly, first classifying each individual observation as “identity affirmed” or “identity threatened” (using the terminology in Bago et al.^49^) depending on their political orientation and what scenario version they had been assigned to. For example, a Republican who responded to the “mask-decrease” scenario was classified as “identity threatened,” because correct interpretation of the data from the fictitious study is likely incongruent with their political worldview. Finally, we used the same classification to test for motivated numeracy (i.e., whether the degree of politically motivated reasoning depended on people’s numeracy).

## Results

**Figure 2:**
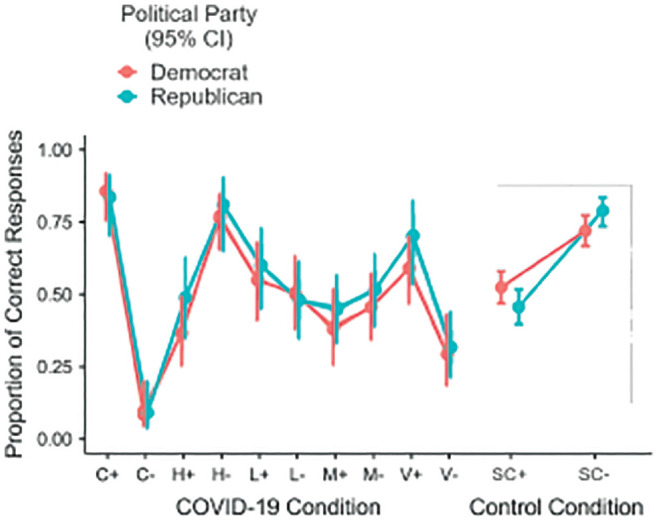
Proportion of correct responses in the different scenarios for Democrats and Republicans. S, symptom; H, hydroxychloroquine; L, lockdown; M, mask; V, vaccine; SC, skin crème. The associated sign stands for the direction of the effect (increase and decrease). Error bars indicate 95% confidence intervals. Sample size: *N* = 1114.

Figure 2 shows the proportion of correct responses for each presented scenario separated by political orientation. As can be seen by the substantial overlap of confidence intervals in the figure, the differences between Democrats and Republicans were small and insignificant in all scenarios, χ^2^(9, *N* = 1114) = 2.16, *P* = 0.99, and there was never a significant difference-in-differences across the 2 versions of the same scenario. Thus, our data are not consistent with politically motivated reasoning about COVID-19–relevant topics. These results remained unchanged when controlling for age, gender, socioeconomic status, and education using logistic models (see Table S1 in the Supplementary Materials) and when restricting the analysis only to the scenario the participants saw first (see Figure S1 in the Supplementary Materials).

Although we found no indication of politically motivated reasoning, there was an overall strong intrascenario difference in correct responses between the two versions (increase v. decrease) for some of the scenarios, in particular the “symptom” scenario but also the “hydroxychloroquine” and “vaccine” scenarios. For example, independently from what the correct response to the task was, that is, averaging the answers across increase and decrease scenarios, 86% of participants interpreted the data in the “symptom” scenario as if dry cough were more present in COVID-19 patients compared with seasonal influenza patients. This implies that most participants (84%) provided the correct answer in the “symptom-increase” scenario but also that most participants (91%) gave the wrong answer in the “symptom-decrease” scenario. Keeping in mind that the data presented in the contingency tables of the different conditions were the same (see Figure 1), our results here suggest that participants’ prior beliefs about the different topics influenced their interpretation of the data shown in the experiment.

To further assess the robustness of our findings and to enable testing for motivated numeracy, we analyzed all COVID-19–polarized scenarios (lockdown, mask, vaccine, hydroxychloroquine) jointly, first classifying each individual observation as “identity affirmed” or “identity threatened” depending on their political orientation and what scenario version they had been assigned to. This means that we incorporated our directional hypotheses for politically motivated reasoning (see the “Materials and Procedure” section above) in the classification of our data. Our result for politically motivated reasoning can be seen in Table 1, model 1, where there is only a minor (and insignificant) increase in the probability of providing a correct response for participants assigned an “identity threatened” scenario, compared with participants in an “identity affirmed” scenario. Thus, again, we found no evidence for motivated reasoning. In model 2, we tested for motivated numeracy, that is, whether politically motivated reasoning is more pronounced (or mainly exists) for some limited range of numeracy. As can be seen in the table, we detected no main effect nor an interactive effect of participants’ numerical abilities. Furthermore, no simple effects analysis disclosed a significant effect considering each scenario separately. Taken together, we found clear evidence against politically motivated reasoning about the effect of COVID-19–related behaviors and policies, and we found no good evidence for motivated numeracy.

**Table 1:** Tests for Politically Motivated Reasoning and Motivated Numeracy Using Joint Data from All COVID-19–Polarized Scenarios^a^

	Model 1	Model 2
Coefficients	ExpB [CI]	ExpB [CI]
Constant	1.68 [1.61, 1.71]	1.67 [1.61, 1.71]
Id-affirmed	0.99 [0.93, 1.05]	0.99 [0.93, 1.05]
Numeracy		0.98 [0.86, 1.12]
Id-affirmed × numeracy		1.05 [0.81, 1.37]
Control variables	Yes	Yes
*N*	880	880

a All models are logistic regressions expressed with the exponential of the odds ratios and Confidence Interval (95% CI) in parentheses. The dependent variable is an indicator variable (=1) for correct response in the scenario to which the subject was assigned. Id-affirmed is an indicator variable (=1) for subjects assigned to a scenario version in which the fictitious data were congruent with their political orientation (=0 instead means that the scenario version was incongruent with their political orientation, i.e., “identity threatening”). Numeracy is the number of correct responses (0–6) from 6 items measuring numeric ability. Control variables include age, gender, socioeconomic status, and education.

## Discussion

At odds with our prestated hypothesis, we found no effect consistent with politically motivated reasoning when interpreting information related to COVID-19. Republicans and Democrats were equally good or bad when interpreting numerical information about the effects of COVID-19–related behaviors and policies. This may seem surprising since the COVID-19 pandemic has been more politically polarizing in the United States than in similar Western countries,^4^ and previous studies have shown a wide range of examples of politically motivated reasoning in polarizing topics related to health care, such as the safety of vaccination, the implied dangers of climate change for public health, and the effects of health care reform.^32–35^ How can we make sense of the fact that we see no effect of politically motivated reasoning in the context of COVID-19 when we see it for other politically polarizing topics?

A plausible, admittedly ad hoc, explanation is that the paradigmatic approach used in much of the literature on politically motivated reasoning (this study included) conflate prior factual beliefs with political group identity and valued beliefs.^36,49,50^ Although prior beliefs and valued beliefs are typically correlated, they are not always the same. The large variation in correct responses across scenarios, despite depicting the same numerical information, was far from random and contingent on the topic of the scenario. Because we find no effect of political group identity on the evaluation of numerical information, this suggests that participants’ prior beliefs about the different topics influenced their interpretation. Thus, rather than motivated reasoning, the partisan divide regarding COVID-19 matters is more likely to be due to lack of reasoning or a form of cognitive indolence. Such interpretation is in line with Pennycook and Rand,^51^ who found that politically motivated reasoning was not a decisive factor when people assessed the plausibility of fake news and that it is laziness rather than deliberate reasoning that can explain why people fall prey to fake news to a different extent depending on their political views.

Furthermore, we found no pattern consistent with motivated numeracy, that is, the hypothesis proposed by Kahan et al.^29^ suggesting that cognitive sophistication or numerical ability is associated with increased polarization when interpreting numerical information about politically and ideological sensitive information. The lack of evidence in support of motivated numeracy is in line with a recent preregistered replication.^52^ Moreover, several recent studies have questioned the idea of motivated numeracy.^4,26,43,53^

On the one hand, our finding that reasoning seems to be primarily driven by prior beliefs and cognitive laziness is good news from a health and science communication perspective. It suggests that most individuals are not deliberately trying to misinterpret or disagree about important health-related information that is politically charged. Thus, as long as people are sufficiently engaged in the matter and actively use their cognitive resources, politically motivated reasoning can be combatted with effective information. However, it is also alarming news because the flood of information from internet and social media that hits people during times such as a pandemic may exacerbate tendencies to not update our beliefs about important health issues in the light of new information.

## Supplemental Material

sj-docx-1-mdm-10.1177_0272989X221118078 – Supplemental material for COVID-19 and Politically Motivated Reasoning Click here for additional data file. Supplemental material, sj-docx-1-mdm-10.1177_0272989X221118078 for COVID-19 and Politically Motivated Reasoning by Allegra Maguire, Emil Persson, Daniel Västfjäll and Gustav Tinghög in Medical Decision Making
