# Supplementary material for: COVID-19 and Politically Motivated Reasoning
Source: Med Decis Making. 2022 Aug 20;42(8):1078–86. doi: 10.1177/0272989X221118078 (PMC9583281; doi:10.1177/0272989X221118078)

**COVID-19 and politically motivated reasoning**

**Supplementary materials**

***S1. Additional analyzes referenced in the results section***

**Table S1. Motivated reasoning in COVID-19 scenarios**

|  | Model 1 | Model 2 |
| --- | --- | --- |
| **Coefficients** | ExpB (SE) | ExpB (SE) |
| Constant | 1.01 (0.07) | 1.03 (0.07) |
| Symptom-increase | 0.85 (0.52) | 0.85 (0.52) |
| Symptom-decrease | 1.1 (0.66) | 1.04 (0.66) |
| Hydroxychloroquine-increase | 1.65 (0.4) | 1.59 (0.4) |
| Hydroxychloroquine-decrease | 1.28 (0.5) | 1.27 (0.5) |
| Lockdown-increase | 1.22 (0.42) | 1.21 (0.42) |
| Lockdown-decrease | 0.93 (0.39) | 0.89 (0.39) |
| Mask-increase | 1.31 (0.38) | 1.33 (0.39) |
| Mask-decrease | 1.27 (0.36) | 1.21 (0.36) |
| Vaccine-increase | 1.63 (0.44) | 1.61 (0.44) |
| Vaccine-decrease | 1.12 (0.41) | 1.07 (0.41) |
| Control variables | no | yes |
| *N* | 1114 | 1114 |

*Simple effects analysis of logistic models showing the difference in proportion of correct responses between Republicans and Democrats for each of the COVID-19 scenarios expressed with the exponential of B coefficients and SE within parenthesis. No significant effect was found.*

**Table S2. Attitudes toward COVID-19 related *behaviours*, by scenario (averaged for increase and decrease versions) and political orientation.**

| Scenario | Democrats | Republicans | *P-value* |
| --- | --- | --- | --- |
| Symptom | 71% | 66% | 0.79 |
| Hydroxychloroquine | 58% | 68% | 0.125 |
| Lockdown | 70% | 53% | 0.013 |
| Mask | 91% | 78% | 0.007 |
| Vaccine | 55% | 42% | 0.062 |

Note: Symptom: Do you feel that coughing in the elbow is the right thing to do to prevent COVID-19 spreading? HCQ: If you were to contract COVID-19, would you like to be treated with Hydroxychloroquine? Lockdown: If your government required a new lockdown, would you follow the restrictions? Mask: Do you feel that wearing face masks in public is the right thing to do? Vaccine: If a vaccine was available today, would you get vaccinated? P-values refer to χ² Tests for each COVID-19 Scenario.

**Table S3. Attitudes toward COVID-19 related *policies*, by scenario (averaged for increase and decrease versions) and political orientation.**

| Scenario | Democrats | Republicans | *P-value* |
| --- | --- | --- | --- |
| Symptom | 99% | 84% | < .001 |
| Hydroxychloroquine | 56% | 66% | 0.16 |
| Lockdown | 85% | 74% | 0.063 |
| Mask | 19% | 42% | < .001 |
| Vaccine | 87% | 76% | 0.033 |

Table 4. Attitudes toward COVID-19 related policies, by scenario (averaged for increase and decrease versions) and political orientation. Following, the presented questions. Symptom: Do you think that social distancing was/is a necessary action to protect the population in your country? HCQ: Do you think that the government should implement a publicly funded policy with Hydroxychloroquine for COVID-19? Lockdown: Do you think that total lockdown was/is a necessary action to protect the population in your Country? Mask: Do you feel that wearing face masks represents a limitation of your freedom? Vaccine: Do you think that the government should implement a publicly funded vaccination policy with voluntary vaccination for COVID-19? P-values refer to χ² Tests for each COVID-19 Scenario.

**Table S4. Tests for motivated reasoning and motivated numeracy using joint data from all COVID-19 polarized scenarios except the vaccine scenario**

|  | *Model 1* | *Model 2* |
| --- | --- | --- |
| ***Coefficients*** | *ExpB (SE)* | *ExpB (SE)* |
| *Constant* | *1.71 (0.02)* | *1.71 (0.02)* |
| *Id-affirmed* | *0.97 (0.04)* | *0.96 (0.04)* |
| *Numeracy* |  | *0.87 (0.08)* |
| *Id-affirmed × numeracy* |  | *0.89 (0.17)* |
| *Control variables* | *yes* | *yes* |
| *N* | *663* | *663* |

*Note: All models are logistic regressions expressed with exponential of the odds-ratios and standard errors in parenthesis. Dependent variable is an indicator variable (=1) for correct response in the scenario the subject was assigned to. Id-affirmed is an indicator variable (=1) for subjects assigned to a scenario-version where the fictitious data was congruent with their political orientation (=0 instead means that the scenario-version was incongruent with their political orientation, i.e., “identity threatening”). Numeracy is the number of correct responses (0-6) from six items measuring numeric ability. Control variables include age, gender, socioeconomic status and education.*

**Fig S1. Proportion of correct responses in the different scenarios for Democrats and Republicans (restricted to first scenario participants saw).**


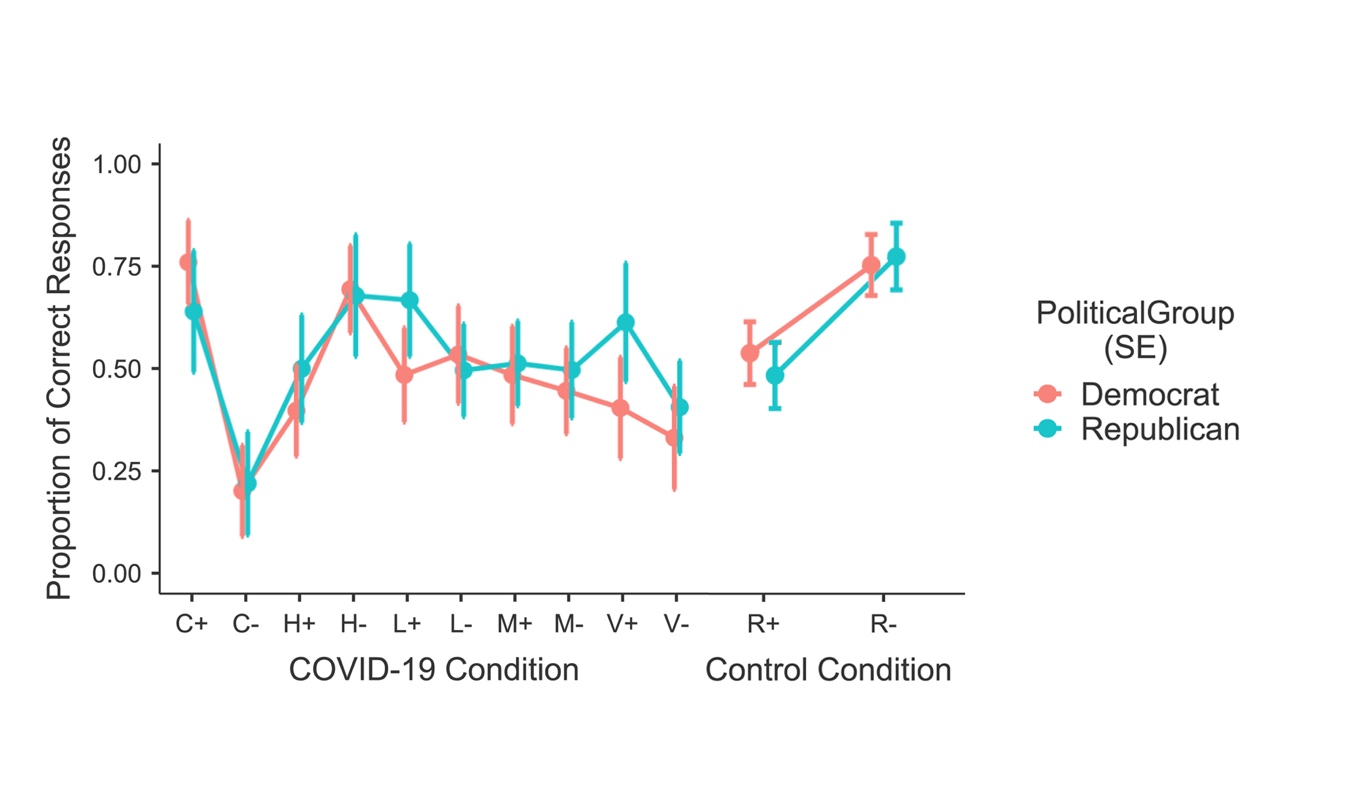

Supplement: sj-docx-1-mdm-10.1177_0272989X221118078 – Supplemental material for COVID-19 and Politically Motivated Reasoning [file sj-docx-1-mdm-10.1177_0272989X221118078.docx]
